# Ultrafast in-line interactive m-mode tool for quantification of left-ventricular (LV) septo-lateral wall motion (SLWM) from high-temporal resolution (6-12ms) cardiac cine steady-state free precession (SSFP) images

**DOI:** 10.1186/1532-429X-13-S1-P37

**Published:** 2011-02-02

**Authors:** Amol Pednekar, Claudio Arena, Janie Swaab, Benjamin Cheong, Raja Muthupillai

**Affiliations:** 1 Philips Healthcare, Houston, TX, USA; 2 Department of Radiology, St.Luke’s Episcopal Hospital, Houston, TX, USA

## Introduction

While conventional temporal resolution on the order of 30-50 ms is sufficient for calculating LV functional metrics such as global ejection fraction (EF), it is insufficient to accurately capture subtle regional wall motion dyssynchrony. A higher frame-rate cine imaging, e.g., 100 fps imposes an undue burden on the manual segmentation, precluding more sophisticated analyses, e.g., measurement of SLWM delays. A robust algorithm that inscribes papillary and trabeculae as LV cavity is a necessary pre-requisite for such an analysis.

## Purpose

To present a high-temporal resolution cine MR imaging method, as well as an ultra-fast, inline M-Mode analysis tool that can measure SLWM delays interactively on the scanner console.

## Methods

All imaging and analysis was done on a standard clinical 1.5T scanner (Achieva, Philips) with a 32channel coil.

### Study Population

16(11m,35±8yrs) asymptomatic volunteers and 10(6m,52±17 yrs) clinical patients.

### MR Acquisition

voxel: 2x2x8 mm3, temporal resolution 5.6-12 ms; TR/TE/flip=2.8-3 ms/1.4-1.5 ms/55deg; time=18 heart-beats/slice, SENSE-factor=3.

### Post-Processing

The processing and analysis steps included: 1) automatic endocardial LV contour delineation for all the cine phases [JCMR 2010;12:O48]; 2) M-mode representation of the LV with overlay of: end-systolic(ES) phase, the onset of peak contraction for septal and lateral wall, and LV centroid.

## Results

**Figure 1:**
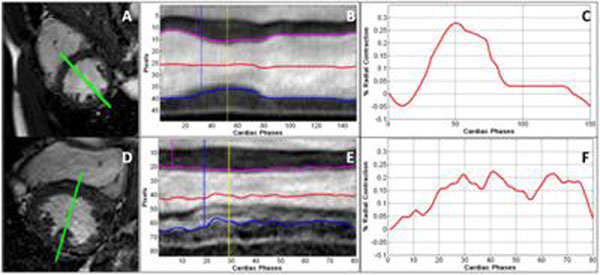
**The normal and abnormal SLWM slice** (A, D) with overlay of septo-lateral line, (B, E) M-mode with overlay of septal (magenta) and lateral (blue) wall contours, and (C, F) percentage radial contraction w.r.t. ED LV diameter. Dotted lines: ES (yellow), and onset of septal (magenta) and lateral (blue) peak contraction. Note the distinct difference in the % radial contraction pattern between the two representative cases.

The user interactively probes the SLWM by prescribing a line, and the corresponding M-mode display is automatically marked with various indices such as time to ES, onset of peak contraction for septal and lateral wall, and percent radial contraction (see Figure 1 and legend for representative results). The total processing time including LV segmentation is less than 1sec per slice for all phases.

## Conclusions

Our results show that LV centroid motion is a less sensitive metric than the measurement of the onset of peak wall motion contraction. Our temporal resolution was sufficient to measure the time difference between the onset of peak contraction for septal and lateral wall (30±31 msec) even in asymptomatic volunteers.

**Figure 2:**
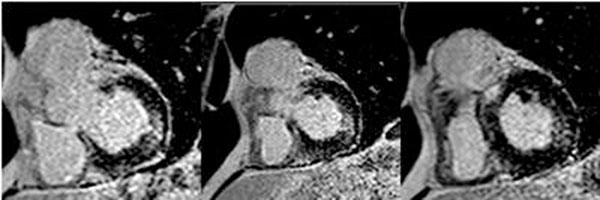
Delayed enhancement images for patient in Figure1D demonstrating a septal and lateral focal scar**.**

